# Understanding Pain in Osteoarthritis: A Clinical Framework Based on the Biopsychosocial Model

**DOI:** 10.1097/TGR.0000000000000504

**Published:** 2026-04-20

**Authors:** Paolo Pedersini, Riccardo Buraschi, Joel Pollet, Rosa Pullara, Massimiliano Gobbo

**Affiliations:** **Author Affiliations:** IRCCS Fondazione Don Carlo Gnocchi, Milan, Italy (Mr Pedersini, Mr Buraschi, Mr Pollet, Mrs Pullara and Dr Gobbo); and Department of Clinical and Experimental Sciences, University of Brescia, Brescia, Italy (Mr Pedersini, Mr Pollet and Gobbo).

**Keywords:** assessment, biopsychosocial approach, osteoarthritis, pain

## Abstract

Patient’s pain experience is a complex phenomenon. The biopsychosocial approach aims to assess pain in all its domains considering: type of pain (nociceptive, neuropathic, nociplastic), psychological, patient beliefs, the socioenvironmental domain, and sensorimotor disintegration. A comprehensive clinical assessment of the patient’s pain experience is helpful to define individual differences between patients and thus to plan effective individualized treatment programs. This paper aims to discuss patients’ pain experience domains based on the biopsychosocial approach in patients with osteoarthritis. Future studies should also consider incorporating a comprehensive pain assessment, which may provide better insight into the role of factors affecting nociception and pain perception.

## INTRODUCTION

The latest Global Burden of Disease Study 2021 confirms that osteoarthritis (OA) has remained a major global public health concern over the past decades. From 1990 to 2020, the number of people living with OA increased by approximately 132.2%, rising from 256 million to 595 million globally. Projections indicate that by 2050, nearly 1 billion individuals will be affected. This growing burden is largely driven by population aging and the rising prevalence of obesity, with high body mass index accounting for 20.4% of OA cases in 2020, up from 16% in 1990. OA has become a leading cause of disability, particularly among adults over 70 years old, where it ranks among the top contributors to years lived with disability.^1^ The discrepancy between the extent of tissue damage and the magnitude of pain, disability, and associated symptoms is a clinical challenge for health care professionals.^2^ The International Classification of Functioning, Disability, and Health view draws on the biopsychosocial model by assessing the person for impairments, activities, participation, as well as environmental and personal factors. The biopsychosocial approach has focused on more general aspects of a person’s health, lifestyle, fitness, and psychosocial domains. Still, the International Classification of Functioning, Disability, and Health view is often misunderstood and applied ineffectively because of a rigid categorization of its domains.^3^ There is substantial variation within individual pain conditions from patient to patient, underscoring the importance of individual assessment. However, several studies in literature have shown that certain pain domains are predictive of poor treatment outcomes in different patient populations.^4^,^5^ These data open new perspectives toward precision medicine-based treatments relying on pain phenotyping as a logical cornerstone principle. The presentations of evaluative approaches and models that include biopsychosocial treatment arise from the clinical need for clustered and directed patient care. In recent years, several researchers have developed and discussed the concept of patient phenotyping, a method of identifying homogeneous subgroups of patients based on relevant prognostic factors. Phenotyping is a clinical and research challenge because it can potentially discriminate pain trajectories and related outcomes.^6^ The Initiative on Methods, Measurement, and Pain Assessment in Clinical Trials, a consortium of pain experts, discussed a call for “precision medicine,” or personalized pain therapeutics. However, before implementing this approach, the characteristics of individual patients or subgroups of patients that increase or decrease the response to a specific treatment need to be identified.^7^ An example of an assessment model that considers different aspects of the clinical experience of pain is presented by Walton and Elliott in 2018.^8^ They discussed an evaluative algorithm by processing a radar diagram showing 7 domains as potential “pain drivers” (nociceptive, neuropathic, nociplastic, psychological, and patient beliefs, socioenvironmental domain, and sensorimotor disintegration). The 7 domains represent different features of a patient’s pain experience, potentially offering greater precision in clinical decision-making than contemporary pain models. The model is presented as a tool to identify the extent of the primary driver(s) of a pain experience without requiring a label on the condition and applies to a wide variety of acute and chronic musculoskeletal conditions. Pain is considered a subjective phenomenon influenced by numerous factors: patient expectations, contextual conditioning, behavioral learning-related experience, and interactions that an individual has with others. Several articles, in support of pain subjectivity, confirm that anatomical impairment is not the predominat reason for the pain experience; additionally, strong evidence shows that the effect of placebo and nocebo contributes to the subjective processing of pain (ie, studies on how contextual factors can change the person’s subjective experience with respect to the perception of a stimulus). This paper discusses patients’ pain experience domains based on the biopsychosocial approach in patients with OA.

## TYPE OF PAIN: NOCICEPTIVE, NOCIPLASTIC, AND NEUROPATHIC PAIN

In recent years, there has been growing recognition that pain in OA involves multiple mechanisms beyond traditional nociceptive and inflammatory pathways, with increasing evidence supporting the role of nociplastic pain driven by central sensitization. Clinicians have considered pain an alarm signal correlated to the intensity of joint degeneration. In OA, most authors have focused their research on local degradation and joint structure, considering pain as only a symptom like a result of joint damage. However, OA pain has a complex pathophysiology, including neuropathic peripheral and central abnormalities and local inflammation involving all joint structures. Recent research emphasizes that it is not a linear condition, that pain experience is independent of structural modifications, and that the quality of pain in OA is important to consider, aside from its intensity.^9^

The term nociplastic pain was adopted by the International Association for the Study of Pain in 2017. Actually, the term nociplastic pain is improperly referred to as synonymous of central sensitization.^10^ Considering the paper by Kosek et al,^11^ who led the task force that approved the term nociplastic pain, it is emphasized that the term nociplastic is used in clinical settings (referring to a symptom) and should be distinguished from central sensitization (which refers to a pathophysiological mechanism related to maladaptive plasticity). This term is very useful to describe pain situations characterized by psychophysical elements that may suggest altered nociception (sensitization), perhaps due to functional or structural changes within the central nervous system (CNS) supported by central and psycho-emotional factors. Clinically, nociplastic pain is described as disproportionate, nonmechanical, diffuse (widespread), with hyperalgesia and/or allodynia, inconsistent stimulus-response pattern, and easy irritability and long-lasting associated burning, cold, tingling symptoms (not neuropathy). Understanding and distinguishing the nociplastic pain pattern holds profound implications, particularly in the context of conditions such as OA.^12^ This distinction is pivotal due to the potential inadequacy of conventional therapeutic interventions in effectively managing nociplastic pain. Consequently, there arises a compelling need to customize treatment methodologies to specifically target the distinctive attributes and underlying complexities of nociplastic pain. This strategic approach is integral to optimizing clinical outcomes and alleviating the challenges posed by nociplastic pain, ensuring a more tailored and efficacious patient care paradigm. Few trials address nociplastic pain due to its recent inception as a term, with the current literature predominantly employing the term “central sensitization.” A recent systematic review about the prevalence of sensitization in patients with knee OA highlights a high heterogeneity in the reported results, mainly based on the diagnostic tool used; however, it has been found a prevalence of 20% of patients with pain sensitization in the considered sample.^13^ Recent studies in patients with OA identified reduced thresholds to pressure pain locally at the affected joint compared to pain-free subjects and similar thresholds at other distant body sites (interpreted as peripheral sensitization),^14^,^15^ while reduced thresholds at the affected joint and at other body sites might be considered a result of proxies of central changes.^16^,^17^ Assessment of nociplastic pain is impactful in patient management. In fact, an interesting study shows that states of pain sensitization are associated with nonresponse after usual care physiotherapy treatment in patients with knee OA.^5^ In the past 5 years, new findings have developed from sensitization studies in patients seen in OA practice.^18^ Additionally, meta-analyses have demonstrated that physical therapy (exercise, manual therapy) and surgical and pharmacological treatment can desensitize the CNS in patients with chronic pain, including patients with OA.^19^-^21^

Although OA pain is traditionally considered nociceptive pain, several studies analyzed neuropathic pain in patients with OA who described it as burning or shooting, common features in neuropathic pain. OA, traditionally considered a degenerative joint disease primarily involving cartilage and bone, is now recognized to have complex pathophysiological mechanisms that extend beyond mechanical factors. Recent studies have indicated that neuropathic pain components might contribute to the overall pain experience in patients with OA.^22^ Nociceptive nerve fibers are thought to become sensitized due to ongoing inflammation and tissue damage associated with OA. One important facet is the potential involvement of neuropathic pain mechanisms. Tekaya et al^23^ highlight a high prevalence (68.3%) of patients with knee OA with neuropathic pain in a cross-sectional study. Additionally, a recent study demonstrated preoperative neuropathic pain and possibly neuropathic pain in 5.6% and 22.2% of the considered patients with OA, respectively.^24^ Individuals with knee OA with suspected neuropathic pain that persisted 6 months post-total knee arthroplasty had higher pain levels, catastrophizing, and depression. Additionally, Yamabe et al^22^ investigated neuropathic pain in patients with hip OA undergoing total hip arthroplasty (THA) using the PainDETECT questionnaire. They found that about one-third of patients exhibited neuropathic or mixed pain prior to surgery, which significantly decreased 6 months after THA, indicating a substantial reduction in neuropathic pain following the procedure. Another interesting cohort study investigated the relationship between the effectiveness of exercise therapy and pretreatment characteristics (radiologic severity, pain sensitization, and neuropathic pain-like symptoms) in patients with knee and hip OA, highlighting the importance of phenotyping patients with OA and its possible impact on treatments effectiveness.^25^ The clinical identification of neuropathic pain in patients with OA remains enigmatic. Given the presence of the 3 types of pain in OA patient populations, clustering patients by type of pain is of fundamental importance for clinical practice and for developing randomized controlled trials targeting specific subgroups of patients.

## PSYCHOLOGICAL DOMAIN AND SOCIOENVIRONMENTAL FACTORS IN PATIENTS WITH OA

The biopsychosocial model provides a comprehensive framework that underscores the complex interplay between physiological and social elements, all of which collectively shape the experience of pain and disability. This paragraph explores into 2 pivotal factors that contribute to the comprehension of OA pain. First, it considers general psychosocial variables that play a significant role in influencing an individual’s pain experience. Furthermore, it explores into pain-specific variables associated with patients’ beliefs, encompassing factors such as catastrophizing, expectations, and pain-related coping strategies. In a recent study, Giotis et al^26^ investigated the impact of knee pain and OA on patients’ quality of life (QoL), focusing on physical, social, and psychological factors. The study found that QoL is more significantly influenced by general factors, such as psychological well-being, pain severity, and overall health status rather than the specific diagnosis of OA itself. This suggests that effective management of knee OA should adopt a rounded approach that addresses these broader factors to enhance patient well-being. The relationship between pain and psychological factors has been demonstrated at multiple levels, spanning from endocrinological responses to intricate CNS dynamics. Investigations into the domain of patients with OA pain have explored stress-related changes in cortisol levels, revealing potential links between the psychosocial stress response and pain perception.^27^ Moreover, the alterations within the pain-specific neuro-matrix have been linked to psychological stress in individuals with OA.^28^ This is reinforced by the demonstrated association between OA pain and mental health, particularly regarding pain flares, as evidenced in prior research.^29^ It is noteworthy that patients grappling with chronic pain conditions, including OA, often report experiencing symptoms of anxiety and depression.^30^ Psychological factors, acting as modifiable determinants, significantly influence the nociceptive process, thereby intricately weaving psychological influences into pain perception. The socioenvironmental domain is characterized by the daily work and family context that a patient is used to experiencing. Latest studies indicate that social factors such as the capacity to describe pain to others, social context, ethnic background, and participation in social activities can influence OA pain and QoL.^31^ Several randomized controlled trial studies suggest that psychosocial interventions can decrease OA pain and disability,^32^ and a recent systematic review highlights a significant association between higher pain scores and psychosocial factors in patients with OA.^33^ Despite the growing evidence regarding the importance of psychological factors in patients with OA pain, these factors are not always considered in conducting trials as variables affecting patients’ pain experience.

## PATIENTS’ BELIEFS IN OA

Patient beliefs are inaccurate or irrational beliefs and thoughts or behaviors about or resulting from the experience of pain. These factors have been extensively studied, categorized, and defined in the literature. A recent paper published by Darlow et al^34^ explored how people make sense of OA through their beliefs, knowledge, and personal experiences, and how these beliefs influence behavior and clinical outcomes. The review found that many people hold impairment-focused, fatalistic beliefs about OA, viewing it as a condition defined by joint damage and inevitable decline, which can lead to disengagement from active self-management and reduced clinician support for non-pharmacological interventions. In contrast, when individuals adopt more dynamic, ecosystem-based understandings of health, where being healthy means being able to participate in meaningful activities, they are more likely to engage in positive lifestyle changes and self-management. The authors emphasize the critical role of clinicians in shaping patients’ beliefs through communication and education, encouraging helpful, adaptive understandings that support participation and well-being. The ones we face most in the literature are catastrophizing, kinesiophobia and fear avoidance belief, illness perception, and self-efficacy. Pain catastrophizing is a maladaptive cognitive-affective response involving exaggerated negative thinking about the pain experience. It has been associated with pain severity and disability in patients with knee OA.^35^ Kinesiophobia and fear avoidance belief are 2 conditions in which the patient has an excessive, irrational, debilitating fear of movement and activity resulting from a feeling of vulnerability to injury or recurrence; fear of moving leads to activity avoidance, and employing strategies to improve these disease-related psychological aspects may be useful in enhancing physical activity participation. Illness perception is the cognitive representations or beliefs that patients have about diseases and medical conditions; recent studies in literature explore the perceptions and experiences of people with knee OA investigating patients’ choice to self-manage their condition and if the perception of the illness affects this choice.^36^ Self-efficacy is the personal confidence the individual has in performing an activity to achieve the desired outcome successfully. Cognitive and behavioral factors also lead to inadequate coping and mistaken beliefs. A recent Cochrane Review about beliefs in people with hip and knee OA supports the concept that patients’ beliefs and chronic pain shape their attitudes and behaviors about how to manage their pain.^37^ Patient beliefs are often factors that substantially influence the clinical outcome of rehabilitation treatments. States of catastrophizing and kinesiophobia have also been shown to underlie sustaining higher pain levels in patients with OA pain. In conclusion, acknowledging and addressing patients’ beliefs, particularly maladaptive ones such as catastrophizing, kinesiophobia, and low self-efficacy, represents a crucial step in optimizing OA pain management. Integrating these cognitive and emotional dimensions into clinical assessment and intervention planning can foster more effective communication, enhance patient engagement in active self-management, and ultimately improve rehabilitation outcomes.

## SENSORIMOTOR DISINTEGRATION

Sensorimotor impairment, or sensorimotor dysregulation, refers to the alteration of the set of all those neurophysiological processes of somatic and sensory nature, implicated in the control of movement, in the regulation of muscle activity, in a broad sense, of all movement control. As with the concept related to central sensitization and nociplastic pain, on the one hand, there is a neurophysiological substrate, measurable under experimental conditions, thus sensorimotor dysregulation, and on the other hand, there is the clinical phenomenon defined as body perception/body awareness. In recent years, research in patients with OA has focused a great deal on the fact that, in the presence of pain, adaptations occur not only at the peripheral level, thus at the musculoskeletal system level, but also at the level of the CNS. These alterations are related to the plasticity of the CNS. It is precisely the peculiarity of the CNS that it learns new strategies to continuously adapt to contextual stimuli. Thus, the phenomena of plasticity at the level of the CNS in patients with pain involve not only the cognitive, emotional, and nociceptive domains, but also affect the somatosensory and motor systems. The motor output, or the movement strategy that the patient generates, is a unique individual strategy.^38^ It is intended to reduce the perception of pain as much as possible and protect the affected body part. The change in motor strategies is also task related, therefore, more related to the individual functional gesture perceived as provocative. In kinematic terms, movement reorganization involves a spectrum of changes, ranging from simple redistribution of activity, both at the level of individual motor units and between muscles (muscle synergies), to complete avoidance of activities or movements. Kinematic output from the motor control system is useful in understanding some variances in current performance and disability in patients with OA. However, as discussed so far, many aspects characterize pain, and few studies to date have considered these aspects in evaluating symptomatic OA.^39^ Motor reorganization does not follow a stereotyped pattern; it is a reorganization based on personal factors (unique and individual), involving several mechanisms that are not mutually exclusive, and thus the importance of considering the subjectivity of the pain experience becomes significant. From a biomechanical perspective, active treatment has been demonstrated to reduce dynamic loading of the knee in patients with OA and potentially promote strategies to treat symptoms and slow down disease progression in OA. However, the definition and assessment of sensorimotor disorders are difficult to standardize and currently applicable much more in a clinical setting. Future studies should aim to obtain assessments of sensorimotor and proprioceptive dysregulation for the purpose of proposing targeted treatments for this alteration.

## IMPLICATIONS FOR REHABILITATION

- A multidimensional pain assessment enables clinicians to identify the predominant mechanisms (nociceptive, neuropathic, nociplastic) and relevant psychological or environmental factors. This supports the design of individualized interventions that are more effective in reducing pain and improving function.
- Integration of psychological and educational interventions addressing emotional dysregulation, maladaptive beliefs, and avoidance behaviors can reduce disability and enhance treatment adherence. Cognitive-behavioral therapy and pain education should be considered essential components of rehabilitation programs.
- Evaluating the patient’s social environment and sensorimotor integration is crucial to achieving long-term functional recovery. Incorporating social support strategies and neuromotor retraining may enhance rehabilitation outcomes.

## CONCLUSIONS

The influence of prevalent pain mechanisms on clinical outcomes in rehabilitation is still being studied. Future studies should also consider incorporating a comprehensive pain assessment, which may provide better insight into the role of factors affecting nociception and pain perception. The biopsychosocial model should be implemented not only in clinical practice but also translationally applied to scientific research to assess all aspects that may characterize the patient’s pain experience. Providing the best available care according to scientific evidence turns out to be the primary goal toward the patient, so the study of an assessment that considers all domains of pain aims as the goal to improve patient-targeted interventions.
